# Finger millet: a hero in the making to combat food insecurity

**DOI:** 10.1007/s00122-024-04637-6

**Published:** 2024-05-21

**Authors:** Hallie Wright, Katrien M. Devos

**Affiliations:** 1 Institute of Plant Breeding, Genetics and Genomics, University of Georgia, Athens, GA 30602 USA; 2 Department of Crop and Soil Sciences, University of Georgia, Athens, GA 30602 USA; 3 Department of Plant Biology, University of Georgia, Athens, GA 30602 USA

## Abstract

Climate change and population growth pose challenges to food security. Major crops such as maize, wheat, and rice are expected to face yield reductions due to warming in the coming years, highlighting the need for incorporating climate-resilient crops in agricultural production systems. Finger millet (*Eleusine coracana* (L.) Gaertn) is a nutritious cereal crop adapted to arid regions that could serve as an alternative crop for sustaining the food supply in low rainfall environments where other crops routinely fail. Despite finger millet’s nutritional qualities and climate resilience, it is deemed an “orphan crop,” neglected by researchers compared to major crops, which has hampered breeding efforts. However, in recent years, finger millet has entered the genomics era. Next-generation sequencing resources, including a chromosome-scale genome assembly, have been developed to support trait characterization. This review discusses the current genetic and genomic resources available for finger millet while addressing the gaps in knowledge and tools that are still needed to aid breeders in bringing finger millet to its full production potential.

## Introduction

Climate change poses a threat to our agricultural systems, especially the production of major crops that currently dominate our food supply, and hence to food security. Crop models for mid- and end-of-century predictions of yields for maize, wheat, rice, and soybean forecast decreases for all four crops caused by warming temperatures, with losses offset to varying extents by yield gains under increased CO_2_ concentrations (Hasegawa et al. 2022). Yield declines are predicted to be most prevalent in low-latitude tropical regions (Jägermeyr et al. 2021), affecting food production and food security in some of the most densely populated and arid areas of the world (Center for International Earth Science Information Network (CIESIN) Columbia University 2018), including in sub-Saharan Africa (van Zonneveld et al. 2023). The grain projected to be most affected is maize. Sub-Saharan Africa, where maize is the predominant cereal grown, has one of the highest population growth rates (Cummins 2019), exacerbating the forecasted food insecurity caused by climate change. India, which became the most populous country in the world in April 2023 and whose population is expected to continue increasing in the coming decades (Hertog et al. 2023), relies heavily on wheat and rice to feed its population. While the predicted global effects of climate change on wheat and rice production are less dire than for maize (Jägermeyr et al. 2021; Zhao et al. 2017)*,* recent studies have projected considerable yield reductions in several wheat and rice growing areas in India (Horie 2019), especially if a reduction in water availability for irrigation during the dry winter (rabi) growing season is taken into account (Daloz et al. 2021). Improving food production in the regions most affected by climate change will require crop diversification (Hufnagel et al. 2020).

Investing in climate-resilient crops that are indigenous to arid regions may be an effective way to sustain the global food supply. One such climate-resilient crop is finger millet, *Eleusine coracana* (L.) Gaertn. Finger millet is a C4 cereal that is predominantly grown by smallholder farmers in eastern Africa, India, and Nepal with minimal inputs under rainfed conditions, thriving even when rainfall is low (Opole et al. 2018). Its grain is high in calcium with protein levels similar to rice (7–9%) although levels as high as 12% have been observed, especially in wild germplasm (Barbeau and Hilu 1993; Virupaksha et al. 1975; J. Zhao and K.M. Devos, unpublished). Finger millet proteins are gluten-free and have a favorable amino acid profile. Levels of methionine, an essential amino acid often lacking in starchy diets, are twofold higher in finger millet than in maize and rice (National Research Council 1996). Its nutritional qualities, adaptation to arid regions, and excellent storage characteristics make finger millet a prime candidate for integration into tropical agricultural systems where traditional crops are under threat. As with other crops grown in these regions, on-farm yields of finger millet are low (1–2 Mg/Ha) (Barbeau and Hilu 1993; Dash et al. 2021; Feyisa et al. 2021; Tenywa et al. 1999; Thilakarathna and Raizada 2015), owing to a combination of factors including management practices, varieties grown, and abiotic and biotic stressors (Odeny et al. 2020). In contrast with maize and sorghum, however, finger millet has undergone very limited improvement. Finger millet has long been viewed as an “orphan crop,” neglected by researchers which, in turn, has hampered breeding efforts. However, finger millet has now entered the genomics era, and genetic and genomic resources are increasingly becoming available. It has also been recognized as an important crop by the Food and Agriculture Organization’s declaration of 2023 as the International Year of Millets. This review is aimed at compiling the current finger millet genetic and genomic resources available to aid in identifying loci for important traits for breeding, while acknowledging the gaps in knowledge and the tools that are still needed to improve its production.

## Origin, domestication, and breeding

**Fig. 1:**
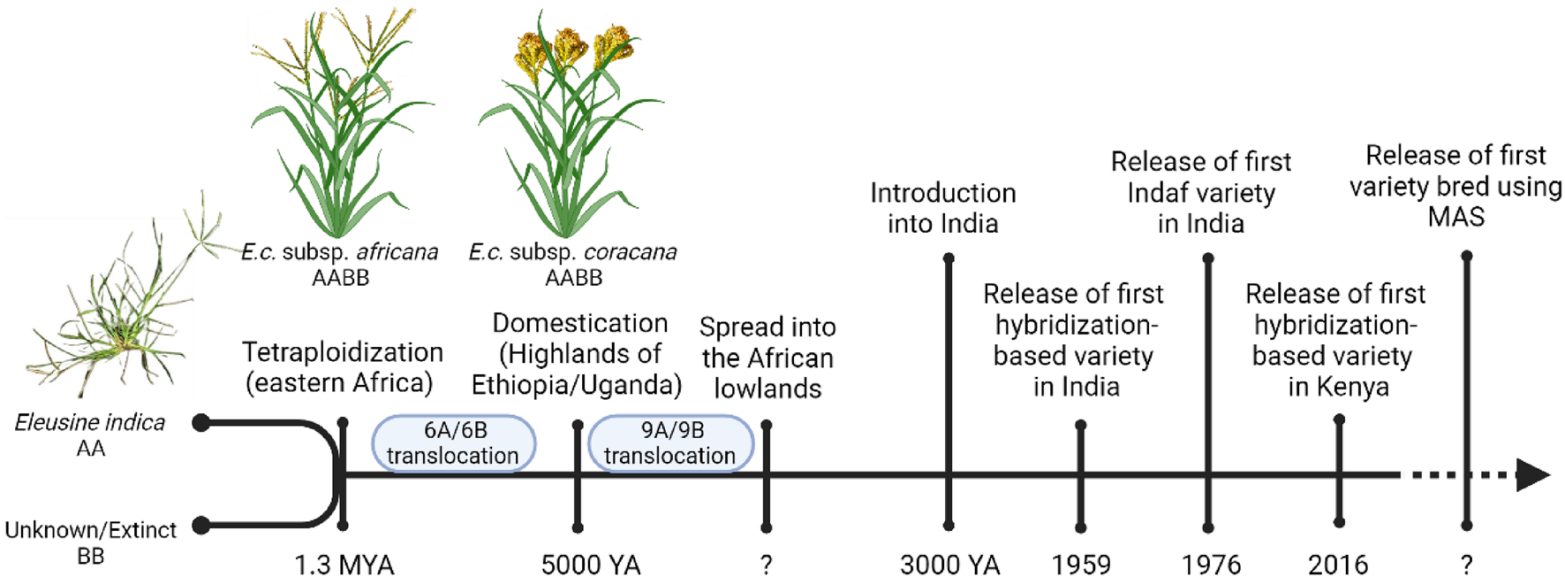
Timeline (not to scale) showing key events in the origin and breeding of finger millet. The figure was created with Biorender.com

Finger millet is an allotetraploid (2*n* = 4*x* = 36, AABB) cereal that belongs to the Chloridoideae subfamily within the grass (Poaceae) family. The maternal AA-genome donor is *Eleusine indica* (wild goosegrass) (Hilu 1988; Hiremath and Salimath 1992), and the BB-genome donor is unknown, likely extinct. Based on the age distribution of genome-specific long terminal repeat retrotransposon (LTR-RT) insertions, the tetraploidization event that gave rise to *E. coracana* (*E.c.*) subspecies *africana* is estimated to have occurred approximately 1.3 million (M) years ago (YA) (Devos et al. 2023) (Fig. 1). Following the tetraploidization event, *E.c.* subspecies *africana* underwent a reciprocal translocation between homoeologous chromosomes 6A and 6B as demonstrated by comparative analyses with *E. indica* (Devos et al. 2023; Qi et al. 2018)*.* Domestication of finger millet from *E.c.* subspecies *africana* took place ~ 5000 YA in the eastern African highlands (Hilu and de Wet 1976; Hilu et al. 1979), marking its primary center of diversity. During or immediately following domestication, a second reciprocal translocation involving chromosomes 9A and 9B took place (Fig. 1). Cultivated finger millet, *E.c.* subspecies *coracana*, then spread from its center of origin in the African highlands to the African lowlands, and subsequently made its way to India (its second center of diversity) through the trade routes ~ 2000 years later (Fuller 2014) (Fig. 1).

Breeding efforts in finger millet (Fig. 1) started some 100 years ago in India and initially consisted of selecting improved lines from locally collected germplasm. Because finger millet is a predominantly inbreeding species, this approach presumably took advantage of new favorable allele combinations generated through occasional natural hybridization events that occurred between finger millet lines planted in close proximity. Introduction of hybridization-based breeding in India in 1951, with the first cross-bred varieties (Purna and Udaya) released in 1959 (Madhusudan et al. N.D.)*,* led to a 50% increase in yield (Joshi et al. 2023). Similar yield improvements were obtained in Kenya with the 2016 release of Maseno 60D (developed at Maseno University) and KACIMMI 42 (developed at the Kenya Agricultural & Livestock Research Organization), the first finger millet varieties developed from targeted crosses in Africa (List et al. 2020; M.M. Dida, personal communication). However, only a small number of breeders in Africa conduct hybridization-based breeding of finger millet. Cross-pollination is difficult because the anthers dehisce before the flower opens (typically between 1 and 5 am) and pollen viability only lasts for about 20 min (Dodake and Dhonukshe 1998). Because of the small size of the flowers, emasculation is typically done by hot water treatment after which the emasculated panicle and a pollen donor panicle are tied together and covered with a bag (Nagaraja et al. 2023; Raj et al. 1984). Successful F_1_ hybrids are often identified using morphological markers, although the use of molecular markers is becoming more prevalent. Further yield increases were obtained by widening the germplasm used in crossing. Hybridization between low-yielding Indian genotypes with higher yielding African accessions resulted in the popular Indaf varieties, the first of which was released in India in 1976 (Joshi et al. 2023) (Fig. 1). The next breeding stage, which has been initiated in India, is to conduct targeted improvement for traits of interest using marker-assisted selection (MAS). The genetic and genomic resources developed for finger millet over the past decade will be key tools in the identification of trait-associated markers that can be exploited to fast-track finger millet improvement.

## Finger millet diversity

As of 2023, Genesys (https://www.genesys-pgr.org/), the portal that provides access to information on global crop germplasm resources, lists over 13,500 finger millet accessions of which 70% are landraces and less than 2% are wild relatives (Genesys 2023). The International Crops Research Institute for the Semi-Arid Tropics (ICRISAT) maintains the largest finger millet collection with some 7500 accessions from 23 countries. A core collection of 622 lines was assembled to represent the geographic regions and the phenotypic diversity of 14 quantitative characters present in the ICRISAT finger millet collection (Upadhyaya et al. 2006). Further phenotypic evaluation of the core collection for 20 morphological traits at five agroecologically diverse locations in India led to the establishment of a mini-core collection of 80 lines (Upadhyaya et al. 2010). The core and mini-core accessions are publicly available and provide a great resource to screen for novel sources of variation for traits of interest.

Genetic analyses of a selection of global *E. coracana* germplasm using several thousand SNP markers show that wild accessions cluster separately from cultivated accessions (Devos et al. 2023; Gimode et al. 2016), indicating limited gene flow between subspecies *coracana* and *africana* despite both species occurring in sympatry in eastern Africa. Low levels of intercrossing between the two subspecies were also suggested by the similar genetic distances of wild accessions to both African and Asian cultivated germplasm (Devos et al. 2023). This is very different from, for example, foxtail millet, where wild and cultivated accessions from the same geographic region intersperse in phylogenetic trees (Thierry et al. 2000; Schröder et al. 2017)*.* Within cultivated finger millet, Indian germplasm is genetically distinct from African germplasm (Devos et al. 2023; Dida et al. 2008; Gimode et al. 2016). That is not surprising, considering that both germplasm groups have remained largely separated since the introduction of finger millet into India around 3000 YA. Germplasm from the center of domestication, Ethiopia, also forms a separate subpopulation (Devos et al. 2023), while accessions from lowland Africa genetically separate in an eastern African subpopulation comprising accessions from, mainly, Kenya, Tanzania, and Uganda, and a southern African subpopulation with accessions from Zimbabwe, Malawi, and Zambia (Bančič et al. 2023; Puranik et al. 2020). Devos et al. (2023) observed that subpopulation membership was largely driven by the presence of wild alleles in pericentromeric regions of specific chromosomes. These regions may be remnants of the domestication process that have persisted for millennia because of the low recombination rates in pericentromeric regions. It is also possible that the wild alleles provide a selective advantage (Devos et al. 2023). Because wild segments are not shared between homoeologous chromosomes, genetic clustering of accessions is different depending on whether genome-wide or subgenome-specific SNPs are used (Bančič et al. 2023; Devos et al. 2023). Overall, polymorphism levels are lower in cultivated compared to wild germplasm due to the domestication bottleneck, and lower in Indian compared to African germplasm due the secondary bottleneck associated with the introduction of finger millet into India (Dida et al. 2008). Introgressing diversity across genetic subpopulations, including from wild accessions, will be essential to improve the productivity and stress tolerance of regionally adapted finger millet. Trait introgressions will be facilitated by the adoption of marker-assisted breeding, allowing simultaneous selection for the trait of interest and elimination of unwanted background effects.

## Genomic regions associated with traits

Although no finger millet cultivar has, as yet, been released that was bred through MAS, the number of traits for which associated markers are available is gradually increasing. Quantitative trait locus (QTL) mapping in biparental populations and genome-wide association studies (GWAS) in panels of diverse accessions provide two avenues for identifying genomic regions and putative candidate genes for traits of interest. Because of the difficulty in generating crosses, trait mapping in biparental populations has been very limited. Pendergast et al. (2021) mapped QTL for flowering date, plant height, panicle number, and blast incidence and severity in F_2_-derived F_3_ progeny from a cross between the cultivated line ACC100007 and an unidentified wild accession. Genotyping was conducted using 5422 SNP markers derived from genotyping-by-sequencing (GBS) (Qi et al. 2018). For all traits, except flowering time which was not examined due to the width of the QTL peak, putative candidate genes were identified, although none were further validated. Utilizing another wild (MD-20) x cultivated (Okhale-1) F_2:3_ mapping population, genotyped with 4400 SNP markers, a QTL controlling purple pigmentation of stigma and anthers that explained 77% of the phenotypic variation was identified (Devos et al. 2023). The causal gene for the purple color trait (*PP*) was determined to be a MYC-bHLH transcription factor orthologous to the maize anthocyanin regulatory gene *R*. Lines with yellow anthers and white stigma had function-inactivating mutations in both the A- and B-genome copies of the MYC-bHLH transcription factor (ELECO.r07.4AG0307750 and ELECO.r07.4BG0338780), while lines with purple stigma and anthers carried a functional A-genome copy and a non-functional B-genome copy (Devos et al. 2023). The same study also determined that two white-seeded lines had function-inactivating mutations in the homoeologous chalcone synthase genes *ELECO.r07.9AG0686670* (nonsense mutation) and *ELECO.r07.9BG0712910* (frameshift mutation). Most likely, knockout of the chromosome 9 chalcone synthase genes eliminates anthocyanin biosynthesis in both the reproductive organs and the seed coat, because these accessions have white stigma and yellow anthers in addition to a white seed coat (Devos et al. 2023). To assist with future trait mapping, some 20 biparental crosses between diverse finger millet lines are being advanced to recombinant inbred lines (RILs), which will greatly increase the capacity in the next few years to conduct QTL mapping in finger millet (M.M. Dida, D.A. Odeny, and K.M. Devos, unpublished).

Several association mapping panels have been assembled for finger millet. Babu et al. (2014) analyzed a population of 190 Indian and African accessions, including the ICRISAT mini-core collection (Upadhyaya et al. 2010), with 104 SSR markers for association with blast resistance. Despite the small number of markers, five QTL for neck and head blast resistance were identified, likely helped by the fact that the SSR markers were derived from nucleotide-binding site leucine-rich repeat (NBS-LRR) genes. Puranik et al. (2020) conducted an association analysis for grain nutritional traits in a different set of 190 accessions that also included the ICRISAT mini-core collection, genotyped with ~ 73,000 GBS SNP markers. A total of 34 significant associations were identified for four minerals, 18 of which were located in genes with homologs involved in binding, remobilization, or transport of metal ions. A drawback of this and other early GWAS studies (Sharma et al. 2018; Tiwari et al. 2020) is the lack of information on the location of the SNPs because SNPs were called de novo from unmapped GBS data. Further, unless very stringent conditions are employed in the stacking of GBS reads during the de novo generation of a GBS reference, homoeologous sequences and paralogs may be grouped together which will conflate intervarietal SNPs identified in a single finger millet subgenome (*i.e.,* SNPs of interest) with SNPs that differentiate the A and B subgenomes or paralogous sequences (Qi et al. 2018)*.* The outcome is a higher-than-expected number of heterozygous SNPs, as was observed by Kumar et al. (2016). Heterozygous SNPs should be rare in an inbreeding species like finger millet. Both the lack of location information and the difficulty in differentiating homologous SNPs from homoeologous SNPs were overcome in more recent studies through use of a chromosome-scale genome assembly (Devos et al. 2023) for GBS read alignment and SNP calling. Dida et al. (2021) identified 19 SNPs associated with blast resistance in a set of 101 accessions comprised of wild relatives, landraces and improved genotypes, and genotyped with ~ 63,000 SNPs generated using Diversity Arrays Technology (DArT) sequencing. The most comprehensive GWAS analysis carried out to date in finger millet involved 423 landraces from Africa and Asia, a total of 8778 stringently filtered DArT-derived SNP markers and 13 phenotypic traits (Bančič et al. 2023)*.* Fifteen genomic regions were associated with three of the traits analyzed. Twelve SNPs were associated with finger length, one SNP with flowering time, and two SNPs with threshing percentage. Use of a common reference genome in SNP calling allows trait locations to be compared across studies. Pendergast et al. (2021) and Dida et al. (2021) both identified a blast resistance locus at ~ 70 Mb on chromosome 6B in a QTL and GWAS study, respectively. Both blast infection studies were conducted in western Kenya, albeit with a time gap of more than 25 years. If the same resistance gene was indeed identified in the two studies, something that cannot be ascertained at this time, this could indicate that the blast fungus did not greatly change over that time period. The genetic stability of the finger millet infecting *Magnaporthe oryzae* lineage in western Kenya is supported by a comparative analysis of blast isolates that were collected from that region with a 20-year gap (K.M. Devos and Sreenivasaprasad, unpublished data). No common trait locations were identified between the Pendergast et al. (2021) and Bančič et al. (2023) studies.

Bančič et al. (2023) is the first study that provides a linkage disequilibrium (LD) estimate for finger millet with *r*^2^ decaying to 0.2 at a distance of 88 kb for the A-genome and 168 kb for the B-genome based on a panel of 423 diverse accessions from Africa and Asia. These values are in line with what has been reported for other inbreeding species (Jaiswal et al. 2019; Novakazi et al. 2020; Rathan et al. 2022). However, LD decay will likely be slower within finger millet subpopulations and breeding pools, which will make it challenging to identify the causal SNPs, and potentially even the causal genes, for traits of interest. While trait-associated markers can be used for breeding, there is always a chance of recombination occurring between the marker and the trait. Causal SNPs, therefore, represent the perfect markers. No studies reported thus far include functional validation of candidate genes, mainly because routine transformation capability is lacking for finger millet. In the absence of functional validation through genetic transformation, the availability of multiple alleles associated with the same phenotype across the A and B genomes, as demonstrated for the anthocyanin locus *PP*, can provide a strong indication of causality (Devos et al. 2023)*.* However, this requires sequence information across diverse accessions or mutant populations which are, as yet, not available for finger millet.

## Whole-genome and transcriptome sequencing resources

Three finger millet genomes have been assembled to date. Hittalmani et al. (2017) conducted Illumina sequencing of the Indian cultivar ML-365, resulting in an assembly with a contig N50 of 24 kb. Hatakeyama et al. (2017) combined Illumina and PacBio sequencing with optical mapping to generate an assembly of the Indian finger millet variety PR202 with a contig N50 of 285 kb and scaffold N50 of 2.7 Mb. An improved scaffold-level assembly of PR202 with a contig N50 of 363 kb and scaffold N50 of 23.9 Mb, generated by the same group, is available from NCBI (https://www.ncbi.nlm.nih.gov/datasets/genome/GCA_021604985.1/). To complement the existing assemblies, Devos et al. (2023) generated a chromosome-scale assembly of the African cultivated genotype KNE 796-S, consisting of nine pairs of homoeologous chromosomes. The initial assembly was generated using 85 × genome coverage of PacBio long reads, scaffolded with 164 × coverage Hi-C, and polished with 95 × coverage Illumina paired-end 150-bp reads. The assembly (*Eleusine coracana* v1.1) with a contig N50 of 15.3 Mb and a scaffold N50 of 61.3 Mb was verified by a high-density genetic map and is available from Phytozome (https://phytozome-next.jgi.doe.gov/info/Ecoracana_v1_1) and from NCBI (https://www.ncbi.nlm.nih.gov/datasets/genome/GCA_032690845.1/). A total of 48,836 high confidence genes were annotated in the KNE 796-S assembly (BUSCO score of 95.2%). The majority of the genes are present in two copies, one on the A-genome and one on the B-genome, as expected for a young (~ 1.3 M years old; Devos et al. 2023) allopolyploid.

**Fig. 2:**
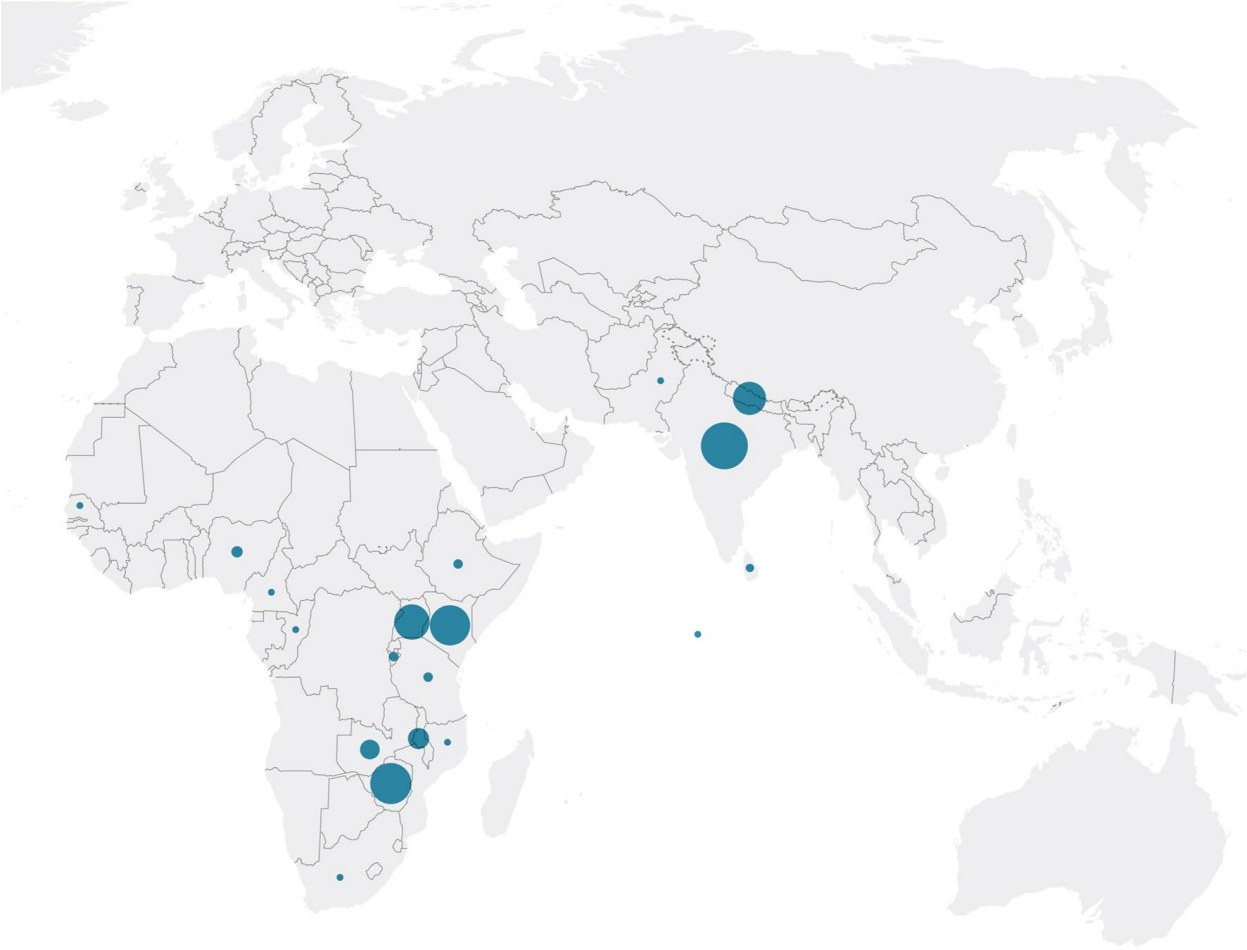
Geographic distribution of the accessions in the finger millet core collection for which country information is available. Finger millet accessions with an unlikely country of origin listing (e.g., Europe) were excluded. The size of the circle correlates with the number of accessions. The figure was created with Datawrapper (datawrapper.de)

Short-read resequencing of finger millet has also been initiated with the aim of capturing the diversity that exists within germplasm collections and to associate polymorphisms with traits of interest. Reddy et al. (2022) sequenced 19 accessions that varied for grain phytic acid (NCBI BioProject PRJDB10731). Devos et al. (2023) resequenced 35 *E.c.* subspecies *coracana*, 11 subspecies *africana,* and two *E. indica* accessions (NCBI BioProjects PRJNA838475 and PRJNA876392). The resequenced lines include the parents of the RIL populations that are under development. Additional sequencing efforts that have, as yet, not been published are ongoing in breeding programs in India (Karnataka germplasm—88 accessions—BioProject PRJNA610152; ICRISAT—finger millet mini-core collection—D. Odeny, personal communication). Accessions sourced from local breeding programs can be limited in their diversity, are sometimes published under in-house names, and may have restrictions in their distribution, which can limit application of the resequencing data even when made publicly available. Therefore, the finger millet community would greatly benefit from a consolidated effort to sequence a set of publicly available accessions that represent the global diversity present in Asian and African finger millet germplasm to accelerate trait discovery. This would preferentially be done using long-read technology to assess the prevalence of copy number (including presence/absence) variation as well as rearrangements in addition to SNP variation. Structural variation can be an important driver of phenotypic variation (Yuan et al. 2021). The finger millet core collection would be a good starting point but needs to be expanded to also include accessions from finger millet growing regions that are currently underrepresented in the collection such as Ethiopia, Tanzania, and Nigeria (Fig. 2). Wild germplasm should also be included in this endeavor to aid in the identification and introgression of alleles that were lost during the domestication bottleneck. Subspecies *africana* is distributed across sub-Saharan Africa (Plants of the World Online; powo.science.kew.org), but is underrepresented in germplasm collections. Only 2% of the *E. coracana* accessions in Genesys belong to subspecies *africana*. To put the lack of resequencing data for finger millet in perspective, over 700 tomato (Gao et al. 2019), 1000 soybean (Valliyodan et al. 2021; Wang et al. 2022a; Yang et al. 2021; Zhou et al. 2015), 2000 maize (Bukowski et al. 2017; Chia et al. 2012; Gore et al. 2009; Grzybowski et al. 2023; Jamann et al. 2017; Jiao et al. 2012; Wang et al. 2020), and 3000 rice accessions (Wang et al. 2018) have been resequenced. Assembling a global fully sequenced finger millet germplasm collection that is held in trust in multiple genebanks around the world for safekeeping and easy distribution will require strong international collaborations as well as participation from the relevant governments. Most, if not all, of the finger millet growing countries have ratified or acceded the Nagoya protocol (https://www.cbd.int/abs/nagoya-protocol/signatories/). Nevertheless, there is legitimate concern about the wrongful exploitation of genetic resources that have not been fully characterized, and hence cannot easily be traced. Whole-genome sequencing of a comprehensive set of diverse germplasm that is made publicly available should ease that concern and ensure that contributing countries will reap the benefits of their participation.

Transcriptome studies in finger millet have also been very limited. RNA-seq datasets are not only valuable for understanding the expression of genes of interest under different conditions, but also for improving gene models and annotation. A total of 17 BioProjects are listed in NCBI SRA (last accessed 01/29/2024) that contain *E. coracana* transcriptome datasets, but some of them lack metadata. Most are unreplicated studies and, while useful for improving the gene annotation of the finger millet genome assemblies, caution should be exerted in employing them for differential gene expression. When assessing gene expression differences between accessions that vary in a morphological trait, pseudoreplication can be used. For example, Brhane et al. (2022) conducted RNA-seq on three aluminum (Al)-tolerant and three Al-susceptible finger millet accessions, and considered the three accessions within each tolerance class as pseudoreplicates. This approach removes a lot of the genotypic variation that is not associated with the trait of interest, similarly to a bulked segregant approach, but requires that the genes and alleles that confer Al tolerance are the same across accessions within a pseudoreplicate. Pseudoreplication has previously been successfully used in the identification of candidate genes for flowering time and wax deposition in switchgrass (Choi et al. 2023; Qi et al. 2021). Another consideration in RNA-seq analyses of allopolyploids is the possible co-alignment of reads from more than one subgenome. The RNA-seq studies in finger millet published to date conducted transcript assembly in the absence of a reference genome (Brhane et al. 2022; Hittalmani et al. 2017; Parvathi et al. 2019; Zhang et al. 2019) or relied on heterologous genome assemblies to be used as a reference (Rahman et al. 2014)*.* Under both scenarios, A- and B-genome transcripts will likely have collapsed to varying extents during read alignments. Combining read counts for the A and B genomes will mask differences in transcript levels that occur in only one of the two subgenomes, a problem that will also occur when aligning reads to allotetraploid finger millet genome assemblies in which the subgenomes are not well resolved. Re-aligning the expression data from these studies to a chromosome-level finger millet genome assembly will improve detection of differentially expressed genes.

## Comparative information

**Fig. 3:**
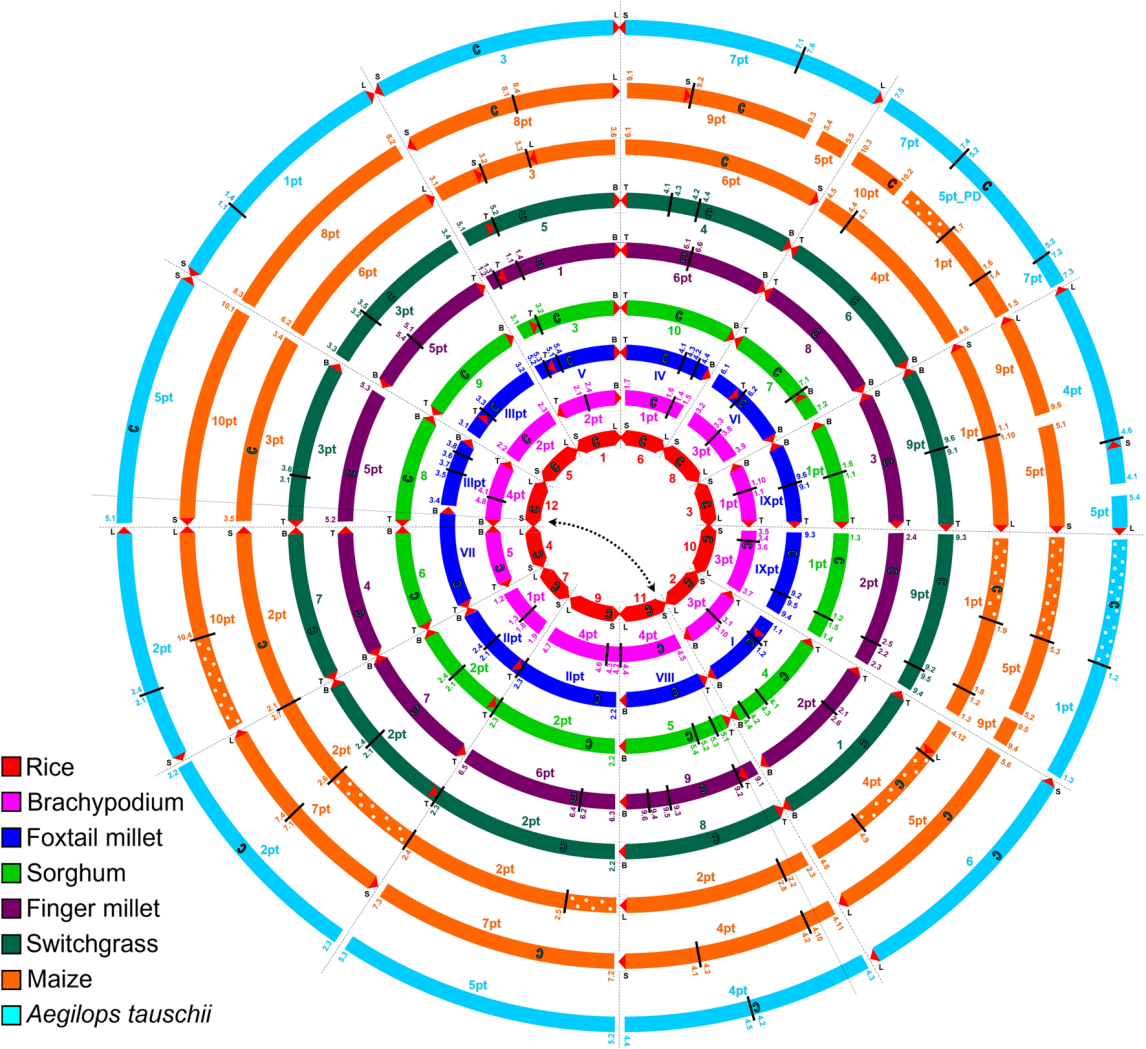
Comparative relationship between finger millet and other grass species. Dotted areas indicate uncertain relationships; red triangles represent telomeres; and short black lines flanked by numbers indicate rearrangement breakpoints with the numbers linking rearranged segments; S, short arm; L, long arm; T, top; B, bottom; C, centromere; and pt, part (of a chromosome) (color figure online)

Comparative information is key to advancing research on under-resourced species especially in the absence of a reference genome. The first finger millet comparative study was conducted at the genetic map level, comparing the chromosomal organization of finger millet and rice (Srinivasachary et al. 2007). With six of the nine finger millet homoeologous chromosomes corresponding to single rice chromosomes, and each of the three remaining chromosomes being syntenic to two rice chromosomes, the finger millet linkage groups were numbered largely in accordance with the rice chromosome nomenclature. The chromosome-level genome assembly allowed these relationships to be refined, leading to the inclusion of finger millet in the grass crop circles (Devos et al. 2023) (Fig. 3). Incorporating genome-based comparative data from orphan crops into available genome relationships allows knowledge from model systems and major crops to be applied to understudied species, but this information flow is not unidirectional. Orphan crops almost certainly carry novel alleles that will lead to the discovery of new gene functions. In demonstration, mapping of seed shattering in finger millet identified a locus that was non-syntenic to the location of previously identified cereal shattering genes (K.M. Devos, unpublished data). A similar observation has been made for shattering in pearl millet (N. Shreshta and A. Doust, personal communication). Although we cannot exclude, at this time, that the non-syntenic locations are caused by small-scale or gene-level rearrangements, the comparative information is suggestive of the involvement of genes not previously associated with this well-studied domestication trait. These observations also underscore that understanding the function of all genes will require linking genotypes and phenotypes across a wide range of species across the tree of life.

## Genetics and genomics resources currently lacking for finger millet

### Mutant populations

Mutant populations can be highly valuable for breeding and for forward and reverse genetics, and have been generated for a range of species, including crops such as maize (Till et al. 2004), rice (Leung et al. 2008; Suzuki et al. 2008; Till et al. 2007; Wu et al. 2005), sorghum (Xin et al. 2008), and wheat (Krasileva et al. 2017; Slade et al. 2005; Uauy et al. 2009). One of the advantages of generating mutations in a polyploid species is that a much higher mutation load is tolerated because of gene redundancy (Comai and Henikoff 2006; Stadler 1929; Uauy et al. 2009). On the other hand, mutations will typically be present in only one of the homoeologous genes and therefore not produce phenotypes, limiting their use for forward genetics. However, when mutant lines are available for each homoeologous gene copy, the “hidden” (no phenotype because of functional redundancy of homoeologous gene copies) mutations can be stacked to generate phenotypes that can be used to study gene functions (Krasileva et al. 2017). Often, these phenotypes are not available as natural variants and may have advantageous characteristics, leading to new varieties (Hazard et al. 2012; Schönhofen et al. 2016)*.* Although a number of studies have investigated the effect of different dose levels of mutagens on finger millet seeds and have identified morphological mutants (Ambavane et al. 2015; Aradhya and Madhavamenon 1979; Eswari et al. 2014; Sellapillai et al. 2022), a comprehensive population of sequenced mutants is, as yet, not available for finger millet. Availability of such populations would dramatically improve trait discovery through reverse genetics approaches. These populations would be especially valuable because transformation is not routine in finger millet, and testing of candidate genes is currently largely limited to using heterologous systems.

## Routine transformation capability

While successful finger millet transformation has been reported, the available systems have, as yet, not been optimized for routine applications. The first study on stable finger millet transformation was published by Latha et al. (2005). Shoot tips were used as ex-plants to generate embryogenic callus that was transformed using a biolistic approach. Bayer et al. (2014) transformed embryogenic callus from seed germinated on callus induction medium using both biolistics and *Agrobacterium*. *Agrobacterium*-mediated transformation has also been reported for callus derived from shoot apical meristem (SAM) tissue (Ceasar and Ignacimuthu 2011). Satish et al. (2017) omitted the callus step and directly transformed and regenerated plants from SAMs, achieving transformation efficiencies as high as 11.8%. However, the published reports on finger millet transformation are few and far between. Furthermore, only Indian cultivars have been tested as a source of ex-plants. Because transformation is often genotype-dependent (Kaur et al. 2022; Wang et al. 2022b), it is unknown how versatile these methods will be across finger millet germplasm as a whole. The recently published leaf transformation protocol (Wang et al. 2023) might provide the breakthrough that is needed to achieve routine transformation in finger millet. Although not yet tested in this crop, the method has been successfully applied in a range of species. In the millets teff (*Eragrostis tef*), foxtail millet (*Setaria italica*), and pearl millet (*Cenchrus americanus*), transformation efficiencies (% transformed T_0_ plants per seedling) of 21, 55, and 7.5%, respectively, were obtained with the maize-optimized protocol (Wang et al. 2023). The leaf transformation protocol circumvents the need for the isolation of immature embryos, which are often used as ex-plants in cereal transformation but are hard to obtain from small millet seeds. Establishment of an efficient transformation protocol will also provide impetus to the development of clustered regularly interspaced palindromic repeats (CRISPR)/Cas in finger millet. Optimization of this technology would be beneficial because cultivars engineered using CRISPR/Cas9 technology have the potential to be exempt from regulations imposed on transgenics. Further, CRISPR screens provide an alternative to the generation of mutant populations for forward genetics, with the added benefit that homoeologous genes can be targeted simultaneously to increase the likelihood of obtaining phenotypes. In summary, there is a vital need for a high-efficiency, genotype-independent, high-throughput transformation system for finger millet to deploy in cultivar development as well as the validation of candidate genes underlying traits of interest.

## Finger millet databases and breeding support

In order for resources to be widely exploited, they need to be publicly available and easily accessible. Germplasm resources for finger millet (13,567 accessions) are accessible through Genesys (https://www.genesys-pgr.org/) (Genesys 2023). As noted earlier, some 55% of the accessions are held by ICRISAT. The second largest holder with 22% is the Genetic Resources Research Institute in Kenya. Overall, 9388 accessions (70%) are listed as “available for distribution” while the status of the remainder is unknown. The Germinate (Lee et al. 2005) Finger Millet Database (https://ics.hutton.ac.uk/cwr/fingermillet/) stores genotypic, phenotypic, and plant passport data, pedigrees, and images but appears, at least for now, to mainly house data generated within select eastern African projects. The current data include germplasm mostly from countries not well represented in Genesys (Fig. 2), as well as trait data and molecular marker data. Milletdb (http://milletdb.novogene.com/) focuses on millets and has capabilities to conduct a range of genomic analyses on the genomes housed by the database (Sun et al. 2023). At the time of writing, only the PR202 finger millet genome (Hatakeyama et al. 2017) was present in Milletdb. A database dedicated to global high-quality finger millet genetic and genomic information is urgently needed to allow data to be incorporated as it is being generated. The database should be breeder-friendly with information on trait locations, associated markers, and, when available, trait variants and primer sequences to screen for the presence of the markers/traits. The finger millet database could be mirrored on existing databases for maize (maizeGDB; maizegdb.org), sorghum (SorghumBase; sorghumbase.org), or the Triticeae (Graingenes; wheat.pw.usda.gov/GG3/). Breeder-relevant information from the millet database could then directly flow into the Excellence in Breeding Platform (excellenceinbreeding.org) to facilitate the implementation of marker-assisted breeding in finger millet.

## Conclusions

Recent events have affirmed the importance for countries to be, at least partially, self-sufficient for food. Under the Russia and Ukraine conflict, transport of nearly 25 million tons of wheat was halted. Although wheat represents less than 15% of the daily food supply in the finger millet growing countries of eastern Africa, many are highly reliant on wheat imports from Russia and Ukraine to fulfill the demand for wheat (Glauber et al. 2023). Further, between March and May 2022, East Africa experienced its worst drought in 40 years (International Federation of Red Cross 2022). Many of the major crops are unable to withstand the adverse effects of current and future climate change. Given unstable political climates as well as climate change, investing in improving locally adapted but understudied climate-resilient crops is critical for maintaining agricultural productivity and achieving self-sufficiency. Finger millet, with its long-term storage ability and high nutritional value, could also serve as a national food reserve for countries in time of crisis.

As the world begins to shift its view on finger millet from a “poor man’s crop” to a “climate-smart super food,” fueling increased interest by urban consumers, finger millet is also starting to gain traction in the research community. The generation of a chromosome-level genome assembly was a first key step to moving finger millet genetics forward. A concerted effort is now needed to build and make publicly available the next set of resources and tools, including mapping populations, fully sequenced global diversity panels, mutant populations, and improved crossing and transformation technologies. Investment in human resource development is also needed. Trait mapping in biparental and GWAS populations will provide trait-associated markers for targeted varietal improvement. However, especially in eastern Africa, there are only a small number of breeders who conduct hybridization-based breeding and hence are able to incorporate marker-assisted trait introgression into their program. Training the next generation of millet breeders in molecular hybridization-based breeding as well as ensuring that they have access to the necessary infrastructure will thus be key to translating the advances in genetic and genomic tools into yield gains.
